# Intersectional Stigma and Multi-Level Barriers to HIV Testing Among Foreign-Born Black Men From the Caribbean

**DOI:** 10.3389/fpubh.2019.00373

**Published:** 2020-01-10

**Authors:** Tonya N. Taylor, Jack DeHovitz, Sabina Hirshfield

**Affiliations:** SUNY Downstate Health Sciences University, Brooklyn, NY, United States

**Keywords:** intersectional stigma, foreign born, Caribbean men, HIV testing barriers, stigma-reducing interventions

## Abstract

Testing is the entry point into the HIV care continuum that includes linkage to and retention in prevention services, and adherence to prevention strategies, including repeat HIV testing. Despite US policy approaches to expand HIV testing to diverse clinical care and community settings, disparities in HIV testing among Black populations persist. Foreign-born (FB) Black persons from the Caribbean have higher annual rates of HIV diagnosis and a higher percentage of late-stage HIV diagnosis, compared with US-born Black persons; and most HIV infections among FB Blacks are among men. In this article, we provide an overview of HIV testing barriers among FB Black men who engage in HIV risk-taking behaviors (e.g., condomless sex with male and/or female partners of unknown HIV serostatus). Barriers to HIV testing for both FB and US-born Black men, include HIV stigma (anticipated, perceived, internalized), low perceived HIV risk, medical or government mistrust, and perceived low access to testing resources. We examine beliefs about masculinity and gender roles that may perpetuate heteronormative stereotypes associated with perceptions of low HIV risk and barriers to HIV testing. We also discuss the impact of recent immigration policies on accessing HIV testing and treatment services and how intersectional stigmas and structural forms of oppression, such as racism, prejudice against select immigrant groups, and homophobia that may further amplify barriers to HIV testing among FB Black men. Finally, we review comprehensive prevention approaches, and suggest innovative approaches, that may improve the uptake of HIV testing among FB Black men.

## Introduction

In the United States (US), foreign-born (FB) persons, defined as individuals who were born outside the continental US and its territories, and naturalized citizens, comprise only 13% of the country's population, yet FB individuals face barriers to accessing health care that may lead to poorer HIV disease outcomes (1, 2). FB persons account for 16% of new HIV infections in the US (2); however, FB Blacks from the Caribbean and Africa have higher annual rates of HIV diagnosis, and late-stage HIV diagnosis, compared to US-born Blacks and FB Whites, thus highlighting disparities due to race and immigration status (3, 4). FB Black men who engage in HIV risk-taking behaviors, including men who have condomless sex with female and/or male partners of unknown serostatus, often reside in metropolitan areas with high HIV prevalence and are less likely to test for HIV than other groups with similar HIV risk behaviors (5, 6).

Universal HIV testing, coupled with regular testing among groups that engage in HIV risk-taking behaviors, is a foundational element of HIV control. Since 2006, the Centers for Disease Control and Prevention (CDC) has recommended universal screening for HIV, and that individuals who engage in HIV risk behaviors should test at least annually, with additional screening as warranted based on risk behavior (7–10). Testing is the entry point to the HIV continuum of care that includes diagnosis, linkage and engagement in HIV medical care, and initiation and adherence to HIV antiretroviral therapy (ART) to achieve viral suppression (10). The UNAIDS, WHO, and the US through the National HIV/AIDS Strategy have set goals for 2020 to improve diagnosis rates among people living with HIV (PLWH), initiate therapy, and achieve viral suppression; all include the goal of having 90% of PLWH aware of their serostatus (11, 12).

Increasing the proportion of persons with HIV aware of their serostatus is the goal of the UNAIDS 90-90-90 treatment target in 2020 (13) but subgroup disparities in HIV testing undermine achieving this goal. For populations with HIV risk-taking behaviors, regular testing is critical, given that most individuals reduce HIV transmission risk behaviors following an HIV diagnosis, that early ART treatment reduces risk of transmission to sexual and drug using partners by lowering viral load, and at an individual level lowers risk for AIDS-defining illnesses and death (14). Additionally, improved screening and detection of HIV at the community level leads to lowered community viral load and a reduced proportion of persons in a potential partner pool with detectable viral load (15), ultimately reducing societal-level suffering and costs associated with HIV/AIDS (16). Despite policy approaches to expand HIV testing to diverse clinical care settings, to implement “opt out” procedures for integrating HIV testing into other standard screening approaches, and to mount community-level campaigns to increase awareness about the importance of HIV screening, disparities in HIV testing among Black populations, especially Caribbean men, persist (17–19).

In Brooklyn, New York (NY), which has the largest Caribbean community in the US, the highest percentage (33%) of new HIV diagnoses are among FB Caribbean individuals, 75% of whom are men (20). New York City (NYC) recently achieved the 90% HIV testing federal goal (21); however, HIV screening among FB Black men with HIV risk-taking behaviors remains suboptimal. Numerous studies have found that late HIV testing is common among FB individuals from the Caribbean (3, 22, 23). Risk factors that adversely affect HIV testing among Black men with HIV risk behaviors, including FB Black Caribbean men, are complex and comprise individual, interpersonal, and structural-level barriers. For example, gender norms surrounding masculinity may impact sexual stereotypes (and associated behaviors) placed on Black men who have sex with men (MSM), along with HIV/STI testing behavior and communication (24, 25). Indeed, culturally oppressive heteronormative gender roles and ideologies perpetuate transmission risk associated with masculinity (26, 27). In addition to oppressive gender norms ascribed to Black men surrounding HIV transmission risk, individual barriers to testing include low perceived risk, endorsing HIV conspiracy beliefs, and perceived low access to testing (26, 27).

Since the inception of the global HIV/AIDS pandemic, there has been a parallel pandemic of stigma and discrimination fueled by blaming the “dangerous infected other” (28). Dr. Jonathan Mann called this the “third level of the AIDS pandemic” (29, 30) and stated that “discrimination is found not only to be [sic] tragic results of the AIDS pandemic, but to be the root societal cause of vulnerability.” HIV stigma, distinct from HIV discrimination, is the ascription of negative attitudes, beliefs and stereotypes about PLWH as undesirable due to fears of contagion, death, or affiliation with “spoiled identity” (31) that diminishes social status, health and wellbeing and produces harmful social consequences (32, 33) and health disparities (33). Fears of loss of privacy, social support and being ostracized due to HIV stigma have had a negative impact on preventive behaviors [e.g., condom use or anti-retroviral therapy (ART) adherence] and has hampered the uptake of HIV testing (34, 35). Internalized HIV stigma has been associated with sexual transmission risk behaviors, including condomless sex (36, 37) and lower likelihood of regular HIV testing (38, 39). HIV screening among FB Black men is influenced by HIV stigma, primarily as it relates to having prejudiced attitudes toward PLWH that perpetuates fears of the social consequences with an HIV diagnosis (40).

Guided by an understanding of intersectional stigma and an ecological model of social determinants of health, this review explores how FB Black Caribbean individuals who inhabit multiple and intersecting stigmatized identities face additional barriers to HIV testing (41, 42). In this review, we also examine gaps in HIV testing initiatives and describe potential novel approaches to lessen barriers and intersectional stigma and develop sustainable HIV testing, treatment, and care programs for this marginalized priority population.

## FB Persons With HIV in the US

FB persons with new HIV diagnoses, compared with newly diagnosed HIV-positive US-born persons, have different group characteristics due to factors related to their immigration pathway, education, health status, local social networks, and the specific risk factors that characterize the global HIV epidemic (5, 23, 43, 44). FB persons with HIV include skilled workers and students pursuing college or graduate training, while other FB persons have become naturalized citizens or immigrate because they have family members that are US citizens or have strong social networks in place in the US (2). The most vulnerable FB individuals with HIV are those that come as undocumented migrants, asylum seekers or refugees who, based on their vulnerable status, have been exposed to political or discriminatory violence due to sexual orientation; some also experience sexual abuse and HIV exposure (45, 46). How vulnerable FB immigrants arrive in the US also impacts how they access health information, insurance, and health care, increasing for some the risk of HIV exposure (47, 48).

Until recently, there was little information about FB Black persons because they were often subsumed under the singular racial category of being “Black.” Ojikutu, Nnaji et al. argued that “All Black People Are Not Alike” in HIV surveillance data and that to explore the epidemiological diversity and more accurately identify HIV prevalence within Black immigrant subgroups, we must disaggregate racial categories by country of birth (23). Thus, grouping US-born and FB Blacks into a single homogeneous racial category masks important sociocultural, linguistic and historical differences that influence health behaviors but also ignores the important impact that migration can have on FB Black's health-seeking behaviors, including HIV testing (49).

## HIV Infection Among FB Caribbean Blacks

HIV disproportionately affects FB Black men in the US who engage in HIV risk behaviors (2–5, 50, 51). In a modeling analysis, the CDC projected that approximately 4,275 FB persons with HIV could immigrate each year to the US (95% CI, 966-5768 persons), assuming that mostly healthier persons would be able to immigrate and that a more accurate prediction may be the lower end of the confidence interval (2, 52). With regard to Caribbean countries of origin, Haiti and Jamaica had the second and eighth highest total number of HIV diagnoses among FB and permanent residents in the US, respectively. California, Florida, NY, and Texas have the highest numbers of FB persons diagnosed with HIV and highest percentages of overall HIV cases in the US (3). In 2016, FB Black individuals from the Caribbean accounted for 22% of new HIV diagnoses in the US, and 70% of those new diagnoses were among FB MSM compared to 75% of US-born men who acquired HIV through MSM contact (2); and 39% of new infections were attributable to heterosexual behavior compared to 27% among US-born persons (2, 3). One study found that Black West Indian men were less likely than were US-born Black men to report non-regular partners (53). Using data from the NYC HIV surveillance registry and American Community survey, another study determined that 61% of FB infections occurred in the US (51); and that the likelihood of the acquisition of HIV in the US was higher for FB males than FB females (68 vs. 50%), and higher for FB MSM (76%), thus highlighting the urgency to promote HIV prevention among new immigrant groups (51).

## FB Black Men and Multi-Level Barriers to HIV Testing

Late HIV testing is common among immigrants from the Caribbean (3, 4, 6, 22, 23, 43). Despite late presentation to HIV care, studies have shown that among FB persons there was no difference in retention in care or virologic suppression compared to US-born persons (54). In the US, factors that account for disparities in HIV screening among Black men with HIV risk behaviors (5, 55–57), including FB Black Caribbean men, are increasingly known (6, 22). Disparities in HIV testing among both US-born and FB Black men with HIV risk behaviors are associated with poor socioeconomic status and greater income inequality (58), poor access to health services (59), decreased access to HIV screening and treatment services (60), delayed health screening (61), and lower engagement with existing social services and related prevention services (62–64). However, numerous studies have noted that differences in HIV testing patterns between FB Black and US-born Blacks are also influenced by nativity (5, 23, 43). Lower levels of HIV testing among FB Black men with HIV risk behaviors, as compared to their US-born male counterparts, is also influenced by differential access to HIV testing and knowledge of how to access testing, lower health insurance coverage (65), and lower familiarity with the US health care system (6). In a recent assessment of 875 Black men with HIV risk behaviors in Brooklyn who reported multiple female partners and condomless sex in the past 6 months, 76% were FB Blacks and only 51% reported HIV testing in the past year (56). Reasons for low HIV testing rates among FB men have included privacy concerns and misconceptions about HIV transmission (66). Another study found that barriers to HIV testing for recent vs. long term immigrants were also due to health access, fatalism and anticipated HIV stigma (6). Among FB Black men with HIV risk behaviors, the benefit of testing more frequently than annually is unknown (67) and warrants investigation, especially since Caribbean men are likely to acquire HIV in the US. Multi-level factors related to immigration status may further undermine FB Black men's access to HIV screening in the US (Table 1).

**Table 1:** Multi-level factors influencing HIV screening among FB Black men with HIV risk behaviors.

**Individual**			**Community**		**Policy**	
• Fear of social consequences of an HIV diagnosis/loss of confidentiality (e.g., discrimination) • Lack of familiarity with US health care system • Distrust of the medical care system and government • Low perception of personal HIV risk and inadequate knowledge about HIV transmission and testing			• High HIV-associated stigma • Prejudice/stereotypes toward PLWH and groups with HIV risk behaviors • Normative distrust in the medical care system • Available testing venues are not advertised/communicated, especially in Creole or languages other than Spanish • Low normative expectations of HIV screening/Gender normative expectations regarding screening • Clinics are female spaces • Real men don't go to doctors		• No HIV testing at US points of entry • No access to Medicaid for 5 years after immigration • Lengthy immigration process • HIV criminalization laws and fears of deportation • Low access to free and confidential/anonymous testing in venues where FB Black men are likely to interface • Few health promotion opportunities emphasize the importance of HIV screening	
**Health outcomes associated with improved screening**	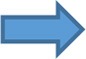	Greater likelihood of viralsuppression; less risk behavior;improved quality and length of life	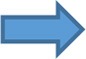	Decreased risk of HIV transmissionto partners	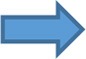	Lower community viralload/proportion withsuppressed viral load

^*^Adapted from the NIMHD Minority Health and Health Disparities Research Framework (68).

### Structural and Policy-Level Factors

A policy-level factor that undermines access to HIV testing for FB Black persons was the removal of HIV testing at US points of entry, which was an unintended consequence of the repeal of the 22-year HIV immigration ban in 2010 (69, 70). The HIV immigration ban, which was first instituted in 1987, prevented non-US citizens with known HIV diagnoses from entering the country (71). The ban permitted the exclusion, removal or deportation of PLWH from the United States. Although the repeal of the HIV immigration ban was considered a watershed moment in eliminating HIV stigma and exceptionalism for PLWH in the US, that repeal of the ban may have inadvertently created more missed opportunities for early diagnosis (69). The repeal of the ban eliminated mandatory HIV testing as part of the physical examination and consequently removed access to free screening at all United States' points of entry. Following the repeal of the ban, the rate of HIV infection among FB individuals rose, especially among persons from the Caribbean. One study found that between 2011 and 2015, the highest rates of HIV infection (8.4%) were among Caribbean men (18).

Two other policy-level barriers to HIV testing for immigrants are restrictions to access Medicaid, and anti-immigrant, anti-Black racism and HIV criminalization laws (72). In the US, legal immigrants are restricted for 5 years after their arrival from accessing federal health benefits, such as Medicaid (73). For the FB Black man unaware of his serostatus, a 5-year delay in access to Medicaid and HIV screening increases the likelihood of HIV transmission. For a FB Black man living with HIV, 5 years without access to antiretroviral therapy could be devastating to his health. The lengthy process for establishing residency or citizenship status to legally work or access health care can further delay access to health care and HIV testing (73). Breanne Palmer (74) argues that anti-immigrant and anti-Black racism in the US legal system has resulted in a disproportionate mass criminalization and deportation of Black immigrants from the Caribbean, compared to immigrants of other races (75). Using public records on US Deportation proceedings in immigration courts, Palmer found that 20.3% of FB individuals deported for committing a crime were Black (74).

Another legal scholar, found that under the illegal NYC “stop and frisk” policy those racially profiled were mostly Black and Latino men, and 98% were deported and 75% of those deportees were male (76). Tanya Golash-Boza (76), whose research focuses on Caribbean populations, found that 10% of deportees in the US were Jamaican, who make up only 2% of legal permanent residents. Police cooperation with Immigration and Customs Enforcement (ICE) in non-sanctuary cities under President Trump's administration has fostered ambivalence and mistrust of the police in Black immigrant communities (77–80). There is evidence that some migrants underutilized HIV testing resources because of misconceptions about immigration laws and that seeking care or a positive test could result in deportation (81, 82). HIV criminalization laws that broadly penalize alleged or perceived non-disclosure of, and exposure to, HIV and non-intentional HIV transmission with incarceration can be an even more detrimental policy-level barrier to HIV screening (83, 84) among FB Blacks because being convicted of a crime can be grounds for deportation. And those deported may likely have even fewer HIV prevention resources available in their home countries.

### Societal-Level Factors

Societal barriers to HIV testing that are specific to FB Black men include anti-immigrant bias, oppressive heteronormative gender roles, and masculine ideologies. Studies have found that xenophobia undermines mental health; however, little is known about xenophobia as a social determinant of health. In an integrative review of the literature, Suleman et al. (85) found that, globally, xenophobia is a “political threat” that can have a deleterious impact on the health and wellbeing of immigrant communities. A well-documented example of stigmatizing or ostracizing a population is the US medical, political, and social response to Haitian-Americans and Haitian immigrants during the 1980s and 1990s when HIV/AIDS was more of a death sentence (86–89). Anti-immigrant rhetoric in the US in the past and present strives to stigmatize and alienate people from other countries. The anti-immigrant racism that resurfaced prior to the 2016 presidential election and became manifest in new discriminatory policies are thought to limit access to health resources and increase racial and ethnic health disparities among immigrant groups and undocumented persons (90). Morey (91) argues that “immigration policy is also ‘health policy”’ and:

“*When immigration policy responds to the worst sentiments of anti-immigrant bias with punitive action, disparity-inducing health consequences follow. When this happens, the vision of Healthy People 2020 of “a society in which all people live long, healthy lives” is compromised. We must recognize how the xenophobic and racist underpinnings of the current anti-immigrant environment contribute to widening health disparities* *(**91**)*.”

Oppressive, heteronormative gender roles and masculine ideologies are key structural barriers to HIV testing among FB Black men (92). Indeed, oppressive beliefs about masculinity, heteronormativity, and heterosexism have been found to be barriers to HIV testing, not only for gay and bisexual men, but also for heterosexual men (39, 93). Gender norms within the Black Diaspora are strikingly similar and create both barriers and facilitators to HIV testing that could include: a man's fear of losing his marriage and ability to provide for his family with a positive HIV test result; fear of being blamed for the spread of HIV; internalized feelings of shame; the perception that clinics are a female space and that “real men” don't go to doctors because it highlights their weakness instead of stoicism and self-reliance; threats to masculinity due to fears that a positive HIV screen might curtail their sexual prowess; and/or being exposed for infidelities, or philandering behavior (94). Mburu et al. argued that HIV stigma “threatened masculine notions of reputation and respectability, independence and emotional control, while it amplified men's risk-taking” and influenced men's participation in HIV services (95). In many African communities, to reveal that one is sick is a stigmatized identity, which extends beyond the individual to the family (96). The stigmatizing sick self is perceived as more severe for men due to the association of sickness as the antithesis of masculine strength (97). Mburu et al. (95) also found that seeking HIV prevention services, or any type of health care, meant that they publicly accepted the stigmatized sick role. Community-level interventions that fostered contact or visibility with the disaffected groups and targeted negative stereotypes have proved effective in reducing HIV stigma and homophobia (98).

### Community-Level Factors

Community-level factors that impact HIV testing for both FB and US-born Black men include HIV-associated stigma (99–103), low normative expectations regarding HIV testing (53, 104), and endorsement of HIV conspiracy beliefs and medical and/or government mistrust (66, 105) that are rooted in historical societal abuses, which create distrust in HIV screening services and higher refusal rates when routine testing is offered (106, 107). However, distinct to FB Black men, there is the challenge of assimilating to US culture and society that may similarly create barriers to access to health prevention screening (108–110), including HIV testing. The process of migrating to another country and cultural system is stressful and often impacts community health outcomes (111–113), including HIV risk behaviors and low HIV screening (114). Losing access to protective social networks are thought to be driving factors of increased vulnerability to HIV infection (115–117). Migrants have fewer economic, cultural and psychosocial resources and often experience difficulty coping with stressful changes of living in another country, which may increase sexual risk behaviors (6). One study found that FB and US-born differences in HIV test-seeking and HIV prevention services are likely due to FB status and the loss of protective, pre-migration sexual networks (49). A study in Canada among Muslim immigrants found that longer time spent in a new country increased knowledge but decreased likelihood of HIV testing (118).

Acculturation has been found to be a key driver in HIV-related sexual behaviors and among immigrants (50, 114, 119–122). A systematic review and subset meta-analysis (119) found that immigrants with high levels of acculturation was associated with condomless sex, sexually transmitted infections (STIs), multiple partners and early sexual debut. They also found that gender moderated the relationship between acculturation, condomless sex, and STIs, and that the relationship between acculturation and condomless sex also varied across different ethnic groups (119). Despite these findings, a key limitation of this study is that most studies examined focused on Hispanics and Latinx samples or female immigrants. Saint-Jean et al. (50) found that the challenges of acculturation in the US and poverty have placed Caribbean-born Black immigrants at higher risk of substance abuse and HIV infection. Despite these challenges, other research has also shown that social integration and political empowerment for some Caribbean communities, including the Haitian and Jamaican communities in NY, has fostered wellbeing (123, 124).

### Individual-Level Factors

Individual-level factors known to lower HIV screening among FB Black men include: lower knowledge of HIV transmission, lower perception of personal or partner HIV risk, reservations or fears about breaches of confidentiality, and potential societal consequences, lack of knowledge about where and how to access testing, speaking a language other than English, low income, no regular provider due to a lack of familiarity with US healthcare system, and recent immigration (6, 125, 126). English-language proficiency is also a known barrier to accessing HIV testing and health care in general (127). Francophone and Haitian immigrants in Brooklyn face greater challenges to accessing health care because of less English fluency and undocumented status (128). Language and cultural barriers can make it harder for many FB individuals to access information about HIV prevention and where to access HIV screening (129). Second, FB Black men are less likely to have a usual source of care or to utilize general preventive care services (130, 131), and are therefore less likely to have routine HIV screening offered, with the exception of men who are currently incarcerated and receive mandatory HIV screening (132, 133). A lack of familiarity with how to navigate the US health care system and system of Medicaid entitlements is another key barrier to accessing HIV prevention and screening services (134).

## Intersectional Stigma

Building on the pioneering theory of *intersectionalities* (135, 136), the framework of intersectional stigma links exposure to oppression with experiences of stigma (137). Individuals with anticipated, perceived, enacted, or internalized HIV stigma often inhabit various marginalized social identities; and have exposure to multiple forms of oppression, known as intersectional stigma. Forms of privilege and oppression intersect with HIV (138, 139), and other stigmatized social identities, such as race and ethnicity, nationality, gender identity, sexual orientation, sexual practices, mental health, and addictions that may increase the cumulative burden of psychological distress (140), contribute to poor clinical outcomes (141) and create disparities in HIV screening (141, 142), especially among Black men (143, 144). However, stigmatized identities have often been analyzed in isolation, ignoring the convergence of intersecting forms of stigma or multiple stigmatized identities that is the reality for many individuals and groups (145). FB Black gay and bisexual men at-risk of acquiring HIV who have low levels of HIV screening (141, 142) may experience layered or intersectional oppression due to racism, anti-immigrant bias, homophobia, and HIV discrimination at work, home, in their communities, as well as while accessing healthcare and HIV screening services.

### Racism: Being a Black, Cis-gender Man

FB Black men experience perceived or anticipated racial or ethnic discrimination in health care settings (61, 146). The influence of perceived racism on HIV testing is not well understood and studies have found mixed results. For example, some studies have found that perceived everyday racism or beliefs in racialized conspiracy theories may improve the likelihood of HIV testing, while other studies have found that perceived healthcare-specific racial discrimination or government/medical mistrust is not a major barrier contributing to the sub-optimal frequency of HIV testing (105, 106, 147, 148). One study found that perceived racism was not inherently detrimental and that it may increase the likelihood of HIV testing and early detection of HIV infection. A clinic-based study in North Carolina found that 90% of the sample had experienced perceived racism and that it was associated with higher odds of HIV testing (147). Alternatively, perceived racism and medical mistrust were found to undermine PrEP awareness and uptake in Black compared to White gay and bisexual men (149). Racial discrimination has also been found to predict lower adherence to HIV treatment (150). One study found that conspiracy beliefs related to pre-exposure prophylaxis (PrEP) were reported more frequently among Black men and transgender women who have sex with men compared to their White counterparts, and that there is an urgent need to address racial medical mistrust so that individuals at risk will understand the potential benefits of PrEP, a highly effective biomedical strategy for HIV prevention (151).

### Racism and Xenophobia: Being a Black Caribbean Man

Anti-immigrant rhetoric before and after the 2016 presidential campaign fueled xenophobic fears that worsened racial and ethnic disparities, especially among Black undocumented immigrants (91). The anti-immigrant campaign slogan, “Build the wall” supported enthusiasm for the implementation of stringent immigration policies along the US-Mexico border and the Executive Order 13769, entitled Protecting the Nation from Foreign Terrorist Entry into the United States, which was also known as the Muslim ban (152, 153). Research suggests that community-level prejudice and xenophobia also increased mortality among immigrant groups (154). Most studies have focused on hate crimes (155–158), especially among transgender and gender-nonconforming individuals (159–161), or police violence and Black Lives Matter before and after the 2016 election (162–165). Seminal studies are now reporting that anti-immigrant policies and sociopolitical stressors are impacting maternal health. Two studies found an association between preterm births among US Latina women and the 2016 presidential election (90, 166, 167).

The global HIV/AIDS pandemic prompted a dubious epidemic of discrimination and racial prejudice. Nations experiencing the pandemic have blamed the “other”—those foreigners or marginalized groups within their society—in order to relocate the source of the deadly contagion safely outside the boundaries of the national identity. In 1988, Dr. Jonathan Mann (30) referred to this process of “shifting the blame” onto the “outsider” as the Third *Level* of the AIDS epidemic. The first level was (and continues to be) the “silent” and undetectable suffusion of HIV infection; followed by the inevitable second tier, which is the visible physical manifestation of the disease syndrome. The third level of the AIDS epidemic is defined as the concurrent epidemic of “blame” and “accusation” (87), in which the “social, cultural, economic, and political reactions to AIDS…[is]… as central to the global AIDS challenge as the disease itself” (30). These social beliefs and fears of contagion, and its causal transmission have resulted in the social control, surveillance, and stigmatization of those infected and afflicted, compromising their right to public health and civil liberties. Today, there is a dearth of research on the intersection of xenophobia, racism and HIV stigma or HIV testing; however, we could speculate that there continues to be an epidemic of blaming the foreign other. More research is needed to explore the impact of anti-immigrant bias on perceived HIV stigma and low HIV testing.

### Homophobia and Racism: Being a Black, Gay, or Bisexual Man

Since the beginning of the HIV epidemic in the US, homophobia has been central to the parallel pandemics of stigma and discrimination (168). However, understandings and experiences of homophobia vary across cultures. Studies have also found a synergism with racism and minority sexual orientation status. Layered or intersecting stigmas due to racism, homophobia, and HIV have made those individuals who are “triply cursed” reluctant to pursue HIV testing (169, 170). In several studies, researchers found that implicit racist and homophobic biases among health care providers may limit access to PrEP among those patients most in need. A study among medical students found that a prevention paradox in their lack of willingness to prescribe PrEP was inconsistent with patient risk and may have been impacted by implicit racial bias (171). Using data from the Urban Men's Health Study, another study found that the combination that racism and minority sexual orientation status has impacts on Black MSM's encountered everyday racism, and thus Black MSM have a less positive experience with the gay community than their White counterparts (172).

A similar study found that Black MSM encounter racism and a less positive experience within the gay community than their White counterparts, suggesting that racism may shape the extent to which gay community affiliation serves as a protective factor against HIV for Black MSM (173). In a qualitative study in Boston (MA) and Jackson (MI), Cahill et al. (149) found evidence that providers and health departments are not adequately addressing medical mistrust among Black gay and bisexual men and other MSM. Black HIV-positive MSM who experienced greater racial discrimination were found less likely to achieve viral suppression compared to Latino HIV-positive MSM (174). Identifying particular sources of perceived racism in health care settings or circulating conspiracy discourses in the Caribbean immigrant community would help to tailor HIV testing strategies for FB Black men.

## Approaches to Reduce Stigma of HIV Testing

UNAIDS recommends that every nation's HIV response should include specifically targeted programs to reduce stigma (175). A review of stigma interventions (176) shows that stigma can manifest on multiple levels, including the intra-personal (e.g., individual-level interventions that aim to reduce the impact of stigma by changing behavior, attitudes, or psychosocial outcomes), interpersonal (e.g., small group-level interventions among individuals who may or may not share the same stigma status, such as HIV serostatus discordant couples), and structural-level (e.g., mass media, change in government or institutional policy). Stigma-focused interventions have generally been delivered in-person; while demonstrating efficacy, these interventions have been implemented on a relatively small scale (177). HIV prevention interventions that include Black MSM have included stigma as an intervention topic (178–180) and a growing number of eHealth interventions have been developed for Black MSM (178, 181, 182) but few interventions have addressed intersectional stigma (169, 183). To date, there are few effective interventions for cis-gender, gay or bisexual FB Black Caribbean immigrant men to promote HIV testing (18). We conducted a review of evidence-based interventions and identified six interventions, ranging from individual to community-level, which target different determinants of HIV prevention and testing, some of which also address stigma.

### Self-Testing

To date, few effective interventions exist for FB Black-Caribbean immigrants to promote HIV testing (18). HIV self-testing via the OraQuick In Home HIV Test does not require interfacing with a clinical setting and has been found to increase self-testing in cohort studies and clinical trials (184, 185). Self-testing for HIV is hypothesized to also reduce the impact of HIV stigma and possibly HIV conspiracy beliefs on testing behaviors (186, 187). However, the prohibitive cost of OraQuick limits its sustainable use in resource-poor settings where many live below the federal poverty line. In addition, the high false-negative rate and inability to detect acute HIV infection are limitations of OraQuick (188). An alternative stigma reduction strategy for HIV self-testing is via Dried Blood Spot (DBS) self-collection kits (189). DBS home collection is currently used for research and clinical purposes to improve access to HIV testing in low-resource settings where access to healthcare and advanced lab techniques are limited (190, 191). DBS self-collection is becoming more common in the US and has been used successfully for self-collecting blood samples for laboratory-quantified viral load among MSM (192). DBS self-collection may become a lower-cost option for collecting blood and mailing the specimen for laboratory-based HIV testing.

### Health Education in the Clinic

Health education interventions can inform both stigmatized and non-stigmatized groups about health issues and reduce perceived stigma by normalizing educational information as part of a general health care visit. A study by Armstrong et al. (193) found that low income Black and Latino men who received the educational intervention reported lower sexual risk and greater sexual health knowledge and health behaviors three months after the intervention. A part of the aforementioned educational intervention is to “teach back,” where the provider teaches the patient health information and then asks the patient to explain what they learned. Healthcare practitioners routinely include “teachable moments” during routine visits to educate patients.

Aung et al. argue that provider-initiated testing and counseling may be the most promising because they foster trust in both the provider and in health systems (82).

### Individual Counseling

Counseling and referral for social support services can be used to provide basic prevention information to screen for unmet needs related to housing, mental health services, and substance use care, and can facilitate navigation to these services (194, 195). These approaches address the HIV screening determinants of HIV transmission and prevention knowledge, address gaps in access to HIV prevention information in this group, and can address gaps in care for unmet service needs. Contact with PLWH is another established and evidence-based strategy to reduce HIV stigma because it can dismantle harmful stereotypes and generalizations that perpetuate fear and misunderstanding (42).

### Active Recall

Brief screening reminders (“active recall”) can promote regular HIV testing (196). In a randomized controlled trial of MSM receiving STI services in Australia, brief automated reminders to engage in testing increased HIV/STI screening to 64% as compared to 30% in a comparison group. A recent review of interventions using this approach revealed no methodologically rigorous evaluations among men with HIV risk behaviors (196). Thus, there is a need to conduct HIV prevention research with FB Black men, and other at-risk populations, to determine the feasibility of automated reminders for health screening. If feasible, this methodology would be cost-efficient (e.g., it is automated and would not require much human effort; the cost per text is low or included in most phone data plans), highly scalable (e.g., most individuals have a mobile phone), and sustainable.

### Male Social Spaces

Barbershops are a natural site for the conduct of health programs on stigmatized issues, including HIV testing. Most FB Black men with HIV risk-taking behaviors are likely to visit a barbershop on a regular basis. In addition, barbershops are typically located in the customer's own neighborhood, allowing public health efforts to focus on prioritization of high-risk geographic areas. Barbershops are trusted community venues for men, and since Black men are less likely than other subgroups to be exposed to more traditional sources of HIV information, such as clinical settings and faith-based organizations (197, 198), they may be more likely to interface with a barbershop than a clinical care setting. Studies have demonstrated the feasibility of partnering with barbershops for HIV prevention efforts, as described in a recent review (198). A recent community-based study in NYC demonstrated success in developing barbershop alliances for recruiting Black men (including 35% FB Black men) for HIV testing programs (199). Thus, partnering with barbershops may be a viable community level approach to increase HIV testing uptake.

### Access to Healthcare Services

Changes in healthcare policy can serve as a structural-level intervention by increasing access to care on a population level. Recent changes in legislation have increased access to healthcare, including the Affordable Care Act (2010), to reduce the number of uninsured individuals. In 2015, the Centers for Medicare & Medicaid Services (CMS) expanded HIV testing coverage to individuals between the ages of 15 and 65 years (200); however, this does not include undocumented immigrants. The Ending the Epidemic (ETE) federal initiative aims to reach groups living with, or at-risk for, HIV with testing and treatment initiatives (201). A recent report by AIDS United (202) provided a framework through which the ETE could effectively reach populations at highest risk of HIV infection, including documented and undocumented immigrants; this included recommended policy changes to include undocumented immigrants in federal HIV testing programs, such as CMS. It is important to note that some changes in healthcare policy have been the result of lobbying by individuals, groups, or corporations, which shows that those who lobby for health care rights can be a catalyst for change in access to services, including HIV testing (203).

### Community-Level Campaigns

Mass media campaigns serve to educate and expose populations to conditions or topics that may be stigmatizing. However, some community-wide media health education campaigns have inadvertently been stigmatizing. A review of US STI prevention and testing mass media campaigns published between 2000 and 2014 cited stigma as the first issue that can impact STI testing and treatment (204). The review found that the campaigns reached 66% of their target populations, on average, and were feasible for reaching at-risk or high-risk populations who may not be reached via the healthcare system or similar services. The authors reported common elements of effective mass media campaigns: obtaining feedback from the target audience to understand the needs of the population and appropriate health messaging; having a theoretically based design; training media staff who run the campaign and interact with the population; reaching the target population with culturally informed messaging through multiple venues such as online, apps, magazine ads, and public transportation billboards; establishing or using existing community partnerships that will provide the services (HIV/STI testing, counseling, etc.) and funds to offer “swag” (e.g., branded condoms, lip balm, t-shirts) in order to legitimize and promote the campaign brand; continual process evaluation to adapt and improve the campaign in real-time; conducting an outcome evaluation to measure the public health impact (e.g., HIV testing rates at specified partner locations before, during, and after the campaign); and continued financial support (via grants or fundraising) to maintain the campaign and its impact. A notable finding from this review was that many of the campaigns did not have rigorous evaluation designs (i.e., no control condition), which is critical to measuring the relative success of a public health campaign.

HIV prevention interventions that include Black MSM have included stigma as an intervention topic (178–180) and a growing number of eHealth interventions have been developed for Black MSM (178, 181, 182), but few interventions have addressed intersectional stigma (169, 183). Stigma-focused interventions have generally been delivered in-person and have been implemented on a relatively small scale (177). In order to address various forms of stigma (e.g., homophobia and racism), on a structural level, stigma-focused interventions must reach much larger populations, as well as collaborate with community coalitions including churches (205, 206). As an example, Frye et al. (207) conducted a rigorous randomized cluster-design trial of community-level intervention on HIV stigma, homophobia, and HIV testing in Black-Caribbean neighborhoods in NYC and found a 350% increase in HIV testing in that neighborhood compared to a control community. Another example of addressing HIV testing on a structural level is by reaching at-risk and high-risk individuals online or via apps. In 2018, Grindr.com implemented an HIV testing reminder system on its site (208) and included HIV status for members to self-identify (e.g., HIV negative, HIV positive, undetectable). Grindr has become as nearly ubiquitous in the MSM community as Facebook is in the general population and could function as an HIV prevention platform, thus reducing stigma associated with testing and with sexual orientation. Similarly, Black MSM and FB Black men may be encouraged to test through mobile apps developed for supportive healthcare interactions and facilitation (209, 210).

### Role of Faith-Based Community

Sanders and Ellen (205) suggest that addressing structural-level issues in communities may reduce racism and poverty more quickly and efficiently. For example, including federal HIV prevention funding for African American community coalitions, including churches, is one way to address racial/ethnic disparities in access to HIV testing and care (205). A review of HIV testing initiatives in Black churches found that, although testing is becoming more available at churches, there is a great need for collaboration between faith-based organizations and public health entities to increase HIV testing and linkage to care (206, 211). Another example to address racial/ethnic HIV testing disparities by involving Black churches is spurring Youth movements through social media; with support from church leadership, Youth movements could help to normalize HIV testing in churches, and consequently reduce HIV testing stigma.

### Gender-Transformative Interventions

Gender-transformative interventions (GTI) “seek to reshape gender relations to become more gender equitable through approaches that ‘free both men and women from the impact of destructive gender and sexual norms”’ (92, 212). Gender within the GTIs is considered as a key social determinant of men's health and has largely focused on understanding masculinity as a set of normative beliefs that is fluid and modifiable. A systematic review of gender-transformative health interventions targeting HIV-related outcomes found 9 out of 11 interventions resulted in statistically significant reductions in sexual risk behaviors and 11 out of 12 violence reduction interventions found statistically significant changes toward gender equality (213). Participants in the pioneering One Man Can Program in South Africa (92) reported greater willingness to pursue HIV testing and fewer concerns about the stigma associated with HIV testing.

## Conclusion

In the US, foreign-born (FB) Black men from the Caribbean with HIV risk-taking behaviors are disproportionately affected by HIV. This disparity, in part, is driven by disparities in HIV screening. Known barriers to HIV testing, such as perceived, anticipated, internalized HIV stigma, low perceived risk, endorsing HIV conspiracy beliefs, and perceived low access to testing are common among FB Blacks. Similarly, established social determinants of sickness and disease that stem from systematic forms of privilege and oppression (e.g., such as poverty, gender inequality, unemployment and limited access to education, quality health care and health promotion information) also undermine the ability of FB Blacks to engage in early testing, care and treatment (3, 4). However, more attention is needed to address the combined impact of layered social stigmas on disparities in HIV screening. And less is known about the links between heteronormative masculine gender roles and ideologies, intersectional stigma and HIV risk. Stigmatized identities have often been analyzed in isolation, but the reality is that intersecting forms of stigma or stigmatized identities seldom operate alone. Caribbean-born, Black gay, bisexual, or heterosexual men living with or at-risk of HIV experience exponential internalized stigma and oppression due to racism, anti-immigrant bias, and homophobia, as well as discrimination at work and in their communities while accessing healthcare and HIV screening services. More culturally responsive research is needed to explore the impact of intersecting stigmas on HIV testing and address the critical gap of HIV risk and poor clinical outcomes across the HIV continuum of care in this priority population.

## Author Contributions

TT developed the topic area and wrote sections of the manuscript. SH wrote sections of the manuscript and provided feedback. JD reviewed and provided feedback on several versions of the manuscript.

### Conflict of Interest

The authors declare that the research was conducted in the absence of any commercial or financial relationships that could be construed as a potential conflict of interest.
